# Main Risk Factors for Ectopic Pregnancy: A Case-Control Study in A Sample of Iranian Women

**Published:** 2014-07-08

**Authors:** Shayesteh Parashi, Somayeh Moukhah, Mahnaz Ashrafi

**Affiliations:** 1 Akbarabadi Hospital, Department of Obstetrics and Gynocology, Collage of Medical Sciences, Iran University of Medical Sciences, Tehran, Iran; 2 Department of Midwifery, Collage of Medical Sciences, Tarbiat Modares University, Tehran, Iran

**Keywords:** Ectopic Pregnancy, Risk Factors, Iran

## Abstract

**Background:**

Although the risk factors of ectopic pregnancy have been determined in previous studies, the main risk factors of ectopic pregnancy are different in various countries due to different cultural and social characteristics. Determination of main risk factors of ectopic pregnancy leads to a rapid diagnosis and an improvement in strategies for its prevention. The purpose of this study was to determine the main risk factors of ectopic pregnancy in a sample of Iranian women.

**Materials and Methods:**

We designed a case-control study to include 150 cases and 300 controls and to compare them by the following factors: socio-demographic characteristics, contraceptive methods, prior tubal surgery, tubal pathology, prior ectopic pregnancy, prior caesarean section, prior abortion, prior infertility, and prior abdominal/pelvic surgery.

**Results:**

The case and control groups were significantly similar in term of education and parity. There was an association between ectopic pregnancy and age which was disappeared after controlling for the main risk factors (adjusted OR=2.45, 95% CI: 0.86-6.97). There was no statistically significant relation between ectopic pregnancy and prior tubal surgery, tubal pathology, prior abortion, prior infertility, assisted reproductive technology, and oral contraceptive method (p>0.05). However, there was a significant association between prior ectopic pregnancy, prior tubal ligation, use of intrauterine device, and prior abdominal/pelvic surgery with ectopic pregnancy (p<0.05). The risk of ectopic pregnancy increased with the use of intrauterine device and tubal ligation, whereas decreased with use of oral contraception.

**Conclusion:**

This study identified prior ectopic pregnancy, prior tubal ligation, use of intrauterine device, and prior pelvic/abdominal surgery as the main risk factors for ectopic pregnancy in a sample of Iranian women. Our findings can be useful for early diagnosis of ectopic pregnancy and for improvement in strategies of its prevention through medical therapy instead of unnecessarily surgical treatment.

## Introduction

An ectopic pregnancy is a complication of pregnancy in which the blastocyst implants anywhere outside endometrial cavity of uterine (1). It is the major cause of maternal mortality during early pregnancy and accounts for 10% of all pregnancy- related deaths. Furthermore, it increases the chances of infertility as well as incidence of the subsequent ectopic pregnancy (2).

Various risk factors for ectopic pregnancy have been identified (2-5) including previous ectopic pregnancy, previous pelvic surgery, induction of ovulation, intrauterine device usage, history of pelvic inflammatory disease (PID), and smoking at the time of conception (2, 6-9). To consider that Iranian women have the certain characteristics (cultural, religious, and traditional values), socio-demographic determinants, sexual behavior and beliefs, and contraception preference, they may have different risk factor profile for ectopic pregnancy compared to women from other countries. Understanding the main risk factors of ectopic pregnancy is valuable due to following factors: rapid diagnosis, less need for surgery, less complications, and an improvement in strategies for prevention of ectopic pregnancy. This study was therefore undertaken to determine the main risk factors of ectopic pregnancy in a sample of Iranian women.

## Materials and Methods

This case-control study was conducted at Shahid Akbarabady Hospital in Tehran, Iran, from March 2006 to May 2011. The data were collected from a total of 300 pregnant women as controls and of 150 case-patient women whose diagnosis of ectopic pregnancy was confirmed by menstrual history, physical examination, serial beta-human chorionic gonadotropin (βhCG), and abdominal/transvaginal ultrasound. There was a history of delayed or skipped menses in women. Initial symptoms were nonspecific, consisting of a period of amenorrhea and abdominal pain or tenderness, with or without unexpected vaginal bleeding. New onset pain was reported, dull or sharp in nature, which was generalized or localized to one area. Furthermore, they complained about spotty or irregular vaginal bleeding. Early physical examination findings included cervical motion tenderness and abdominal tenderness on abdominal palpation.

All data were collected from the hospital’s medical records, while one woman in case group was compared with two women in the control group at the same time. A questionnaire completed by the corresponding author for the patient in case group involved demographic characteristics, menstrual history, present pregnancy status, diagnostic actions, and management, whereas for women in control group, the last two items, i.e. diagnostic actions and management, were omitted from questionnaire.

Several studies (9-12) have categorized the intensive risk factors as follows: prior tubal surgery, prior tubal pathology, and prior ectopic pregnancy, while infertility as moderate risk factor and prior abdominal/pelvic surgery as low-risk factors have been considered for incidence of ectopic pregnancy. Similarly, in the current investigation, such risk factors were categorized, analyzed, and presented.

### Statistical analysis

We used Statistical Package for Social Science (SPSS; SPSS Inc., Chicago, IL, USA) version 16 for data analysis. Odds ratio (OR) and 95% confidence intervals (CI) obtained after binary logistic regression were used to describe the association between ectopic pregnancy and potential risk factors. Multivariable modeling was employed to determine which factors were associated with ectopic pregnancy, while the association was adjusted for other variables. In this model, we only included variables that were associated with the risk of ectopic pregnancy in univariate analysis.

### Ethical considerations

Our study was a retrospective study including patient files, and we did not directly contact with patient, but our study was confirmed by Ethical Committee of the Shahid Akbarabadi Hospital.

## Results

The data for socio-demographic characteristics (age and educational level), parity, and abortion of women in cases and controls are presented in table 1. The average age of women in case and control groups was 28.7 ± 6.0 years (range 16-44 years) and 26.4 ± 5.6 years (range 17-46 years), respectively. The association between ectopic pregnancy and age (Table 1) disappeared after controlling for the main risk factors (Table 2). The case and control group had similar parity as well as educational level (Table 1). The common chief compliance in women with ectopic pregnancy was bleeding and pain.

**Table 1:** Comparison of age, educational level, parity and abortion of subjects between case and control groups from March 2006 to May 2011


	Case(n=150)	Control(n=300)	Crude OR	95% CI
	N (%)	N (%)		

**Women´s age (Y)**				
**16-20**	10 (6.7)	49 (16.3)	0.36	0.18-0.74
**21-25**	35 (23.3)	102(34.0)	0.59	0.37-0.92
**26-30**	50 (33.3)	78 (26.0)	1.42	0.92-2.18
**31-35**	31 (20.7)	51 (17.0)	1.27	0.77-2.09
**≥ 36**	24 (16.0)	20 (6.7)	1	-
**Educational level**				
**0-8**	79 (52.7)	172 (57.3)	0.88	0.29-2.71
**9-12**	55 (36.7)	87 (29.0)	1.38	0.44-4.27
**13-18**	7 (4.7)	12 (4.0)	1	-
**missing**	9 (6.0)	29 (9.7)		
**Parity**				
**0**	56 (37.3)	152(50.7)	1.14	0.54-2.4
**1**	61 (40.7)	91 (30.3)	1.30	0.66-2.57
**≥ 2**	33 (22.0)	57 (19.0)	1	
**Abortion**				
**0**	66 (44)	257 (85.7)	0.13	0.08-0.21
**1**	63 (42)	32 (10.7)	6.33	3.87-10.35
**≥ 2**	21 (14)	11(3.7)	1	-


The comparison of contraceptive methods among the two groups is shown in table 3. A total number of 34 (22.7%) patients in case group and 14 (4.7%) subjects in control group used intrauterine device (IUD) at the time of conception. The oral contraceptive method was recorded in 8 (5.3%) and 24 (8.0%) subjects of case and control groups, respectively. The usage of tubal ligation (TL) was observed in 12 (8%) and 5 (1.7%) women of case and control groups, respectively. Overall, it was revealed that the usage of IUD and TL significantly increased risk of ectopic pregnancy (adjusted OR=4.79, 95% CI:1.61-14.21 and adjusted OR=4.49, 95% CI:1.16-17.28, respectively), whereas oral contraceptives decreased risk of ectopic pregnancy (adjusted OR=0.7, 95% CI:0.23-2.15) (Table 2).

Table 3 presents the association of three categories of risk factors (high, moderate, and low) with incidence of ectopic pregnancy. It was observed that the risk factors of ectopic pregnancy in women are previous tubal surgery, tubal pathology, assisted reproductive technology (ART), sterilization, previous ectopic pregnancy, IUD usage, infertility, and abdominal/pelvic surgery.

**Table 2:** Main risk factors of ectopic pregnancy after final logistic regression analysis (random effects model)


	Adjusted OR	95% CI	P value*

**Age (Y)**	2.45	0.86-6.97	0.09
**Abortion**	1.08	0.36-3.25	0.88
**Tubal surgery**	0.45	0.05-3.91	0.47
**Tubal pathology**	0.3	0.01-8.04	0.47
**ART**	1.93	0.15-23.43	0.60
**Abdominal/Pelvic surgery**	5.24	2.04-13.44	< 0.001
**Ectopic pregnancy**	57.93	6.79-494.25	< 0.001
**Infertility**	1.92	0.4-9.09	0.40
**Oral contraception**	0.7	0.23-2.15	0.54
**TL**	4.49	1.16-17.28	< 0.001
**IUD**	4.79	1.61-14.21	< 0.001


IUD; Intrauterine device, TL; Tubal ligation, ART; Assisted reproductive technology, CI; Confidence interval and *; For variables with more than two categories, the p value of test for trend is given.

**Table 3:** Contraceptive methods and risk factors of ectopic pregnancy compared between subjects of case and control groups


	Cases(n=150)No (%)	Control(n=300)No (%)	CrudeOR	95% CI

**Contraceptive methods**				
**IUD**				
**No**	116(77.3)	286(95.3)	1	
**Yes**	34 (22.7)	14 (4.7)	5. 98	3.09-11.57
**Oral Contraception**				
**No**	142 (94.5)	276 (92)	1	
**Yes**	8 (5.5)	24 (8.0)	0.64	0.28-1.47
**TL**				
**No**	138 (92)	295 (98.3)	1	
**Yes**	12 (8)	5 (1.7)	5.13	1.77-14.84
**High Risk Factors**				
**Tubal Surgery**				
**No**	100 (96.7)	296 (98.7)	1	
**Yes**	/5 (3.3)/	4 (1.3)	2.55	0.67-9.64
**Tubal Pathology**				
**No**	147 (98)	297 (99)	1	
**Yes**	3 (2.0)	3 (1)	2.02	0.4-10.13
**ART**				
**No**	196 (97.3)	300 (100)	1	
**Yes**	4 (2.7)	0	2.02	0.4-10.13
**Ectopic Pregnancy**				
**No**	117 (78)	299 (99.7)	1	11.4-623.69
**Yes**	33 (22)	1 (0.3)	84.33	
**Moderate Risk Factors**				
**Infertility**				
**No**	136 (90.7)	293 (97.7)	1	
**Yes**	14 (9.3)	7 (2.3)	1.75	0.44-6.96
**Low Risk Factors**				
**Abdominal/Pelvic surgery**				
**No**	101 (67.3)	292 (97.3)	1	
**Yes**	49 (32.7)	8 (2.7)	17.70	8.11-38.66


IUD; Intrauterine device, TL; Tubal ligation, ART; Assisted reproductive technology and CI; Confidence interval

The main risk factors of ectopic pregnancy are shown in the table 2. Prior ectopic pregnancy, prior tubal ligation, use of IUD and prior pelvic/abdominal surgery were identified as four main risk factors mentioned in obstetric history. Prior ectopic pregnancy was associated with an increased risk of ectopic pregnancy (crude OR=84.3, 95% CI: 11.4-623.7). Adjustment did not affect this relationship (adjusted OR=57.9, 95% CI: 6.79-494.2; Table 2). There was no significant difference between case and control groups with regard to the prior tubal surgery (adjusted OR=0.45, 95% CI: 0.05-3.91), and tubal pathology (adjusted OR=0.3, 95% CI: 0.01-8.04). There was no statistically significant association between ART (adjusted OR=1.93, 95% CI: 0.15-23.4), infertility (adjusted OR=1.92, 95% CI:0.4-9.09) and ectopic pregnancy (Table 2). Prior abdominal/pelvic surgery was associated with an increased risk of ectopic pregnancy (crude OR=17.7, 95 CI: 8.11-38.7). After adjustment for the main risk factors, the association persisted (adjusted OR=5.24, 95 CI:2.04-13.4; Tables 2 and 3).

## Discussion

In the current investigation, we demonstrated that the risk of ectopic pregnancy increases in women over 30 years of age. Similarly, in a case-control study by Karaer et al., they have found that an increase in rate of ectopic pregnancy coincides with an increase in the age of women before reaching age 40 (13). Bouyer et al. in a large case-control study in France also reported a significant relation between age and ectopic pregnancy (6).

The role of age in the incidence of ectopic pregnancy has been suggested by researchers. However, studies have produced conflicting results in this respect (3, 5, 14-18). Thus, the precise physiological impact of advanced maternal age on ectopic pregnancy risk is unclear (6). It is highly improbable that an increase in chromosomal abnormalities in the trophoblastic tissue be casued by advanced maternal age (19, 20). It has been reported that the age-related changes in tubal function may delay ovum transport, leading to tubal implantation. However, these hypotheses remain to be tested. Furthermore, Coste et al., (1991) found that this association refers to the probability of exposure to most risk factors which increases with age (3). Conversely, another study has suggested that age plays a more important role as compared to other risk factors (21).

In this study, we found that there is a relation between use of IUD, oral contraception and TL with risk of ectopic pregnancy. These observations are similar to those previously reported by Chow et al., (1987) (2). The usage of contraceptive methods, i.e. IUD and TL, increases significantly risk of ectopic pregnancy, whereas oral contraception prevents ectopic pregnancy. This may be due to the fact that the oral contraception prevents pregnancy through inhibition of ovulation. Mol et al. (22) in a meta-analysis found that women becoming pregnant while using an IUD are at increased risk of ectopic pregnancy. In a well-designed case-control study, Bouyer et al. found that IUD may have an etiological role in occurrence of ectopic pregnancy (6, 23). In another case-control study in Turkey, similar results were found (13). IUDs does not prevent ovulation, so it is more effective for preventing intrauterine pregnancy than extra-uterine pregnancy, indicating the increased risk of ectopic pregnancy.

A Danish study found that 76% of post sterilization pregnancies were ectopic (24). A large case-control study reported "a 10-year cumulative probability of ectopic pregnancy for all sterilization methods of 7.3 per 1000 procedure" (25). The US Collaborative Review of Sterilization (CREST) study, a large multicenter prospective study, reported a rate of 32.9% for ectopic pregnancy among post tubal ligation (TL) procedure (26).

The results of the present study showed there is no relation between prior abortion and prior infertility with incidence of ectopic pregnancy. The results obtained about association of prior abortion and incidence of ectopic pregnancy were in agreement with those previously achieved by Ankum et al. in a meta-analysis study, in which they observed no significant association between spontaneous abortion and risk of ectopic pregnancy (OR=0.63-1.5) (9), while Bouyer et al. have found a strong relation between abortion and incidence of ectopic pregnancy, especially in women with three or more spontaneous abortion (6). Similar inconsistent findings have also been reported about the association of induced abortion and incidence of ectopic pregnancy. For example, Coste et al. (20) suggested that induced abortion may be a risk factor for ectopic pregnancy, whereas other studies have reported no significant association between induced abortion and ectopic pregnancy (27-29).

In a follow-up analysis by Tuomivaara and Ronnberg (30), they have evaluated 929 infertile couples regarding ectopic pregnancy. Their findings showed the rate values of 46% and 9% for conception and ectopic pregnancies, respectively, indicating strongest association between a current ectopic pregnancy and a previous ectopic pregnancy (9.9-fold risk) as compared to women with primary infertility. In a meta-analysis study by Ankum et al. (9), they reported an increased risk of 2.5-23-fold for ectopic pregnancy, suggesting an association with a history of infertility.

Similar to our findings, other studies also found an increased risk of 7-9-fold among women with a history of prior ectopic pregnancy (12). Karaer et al. also found a strong relation between previous ectopic pregnancy and ectopic pregnancy. They concluded that a woman with a damaged fallopian tube or another intrinsic factor leading to a previous ectopic pregnancy has a greater tendency toward a subsequent ectopic pregnancy (13).

We found that there was a significant relationship between risk factors like abdominal/pelvic surgery and incidence of ectopic pregnancy. In a meta-analysis study of Ankum et al. (9), they reported that the majority of experiments had found a range of OR from 0.93 to 3.8 for pelvic or abdominal surgery. The observed association between pelvic surgery and ectopic pregnancy may be explained by peritoneal and peritubal adhesions that often occur with this type of surgery.

We found that there is no significant relationship between risk factors such as prior tubal surgery, tubal pathology, ART and incidence of ectopic pregnancy. Similarity, Karaer et al. (13) did not find any association between prior tubal surgery and ectopic pregnancy, which was due to the low number of subjects in their study. However, Bouyer et al. (6) showed that the tubal surgery is the most important factor for incidence of ectopic pregnancy. In a large population-based database of pregnancies conceived with ART in US clinics between 1991 and 2001, 2.1% were ectopic (31).

Tubal pathology referred as prior tubal infection is also found to have a probable causal relation with ectopic pregnancy. In Sweden, a decrease in the rate of chlamydial infection led to a reduction in the incidence of ectopic pregnancy (16). In addition, Bouyer et al. (6) reported that infectious factors and tubal surgery are the most important risk factors for ectopic pregnancy. In the current investigation, we could not determine the association between the incidence of ectopic pregnancy and some parameters like history of smoking and pelvic infection because of socio-demographic characteristics of Iranian women.

## Conclusion

Since incidence of ectopic pregnancy is likely to be considered as an important role for future fertility, we designed this study to identify the risk factors of ectopic pregnancy in a sample of Iranian women. In the present study, we found that the main risk factors for incidence of ectopic pregnancy in a sample of Iranian women are prior ectopic pregnancy, prior tubal ligation, usage of IUD, and prior pelvic/abdominal surgery. In addition, ectopic pregnancy was positively related to the previous history of ectopic pregnancy, abortion, caesarean section, and infertility. These findings can be useful for early diagnosis of ectopic pregnancy to pursue proper medical therapy instead of unnecessarily surgical treatment.
